# Remifentanil for Carboprost-Induced Adverse Reactions During Cesarean Delivery Under Combined Spinal-Epidural Anesthesia

**DOI:** 10.3389/fphar.2020.00980

**Published:** 2020-06-30

**Authors:** Chang-na Wei, Xiang-yang Chang, Jin-hua Dong, Qing-he Zhou

**Affiliations:** ^1^ Department of Anesthesia, Jiaxing University Affiliated Women and Children Hospital, Jiaxing, China; ^2^ Department of Obstetrics, Jiaxing University Affiliated Women and Children Hospital, Jiaxing, China; ^3^ Department of Anesthesia, Affiliated Hospital of Jiaxing University, Jiaxing, China

**Keywords:** remifentanil, carboprost, adverse reaction, cesarean delivery, intraspinal anesthesia

## Abstract

**Purpose:**

Carboprost may induce adverse reactions when used to treat postpartum hemorrhage. We aimed to explore the effects of intravenous infusion of low-dose remifentanil to prevent such reactions.

**Methods:**

We enrolled parturient patients scheduled for elective cesarean section. Anesthesiologist administered combined spinal epidurals at the L3/4 interspace, with 0.5% hyperbaric bupivacaine subarachnoid space injections (1.5–2.5 ml). We randomly divided parturient patients, administered carboprost during surgery, into the remifentanil group (group R) and the control group (group C). Patients in group R received an intravenous target-controlled infusion of remifentanil (target effect-site concentration, 1.5 ng/ml) simultaneously with a carboprost tromethamine injection (250 µg). Patients in group C received a normal saline infusion with carboprost. We recorded and analyzed the incidence of carboprost-related adverse reactions (vomiting, nausea, chest congestion, flushing, hypertension, tachycardia, cough, and shivering), and assessed patient comfort using a numerical rating scale ([NRS], on which 0 was very uncomfortable and 10 was very comfortable).

**Results:**

After applying inclusion and exclusion criteria, we conducted statistical analysis of the data from 70 women. The incidence of vomiting was significantly lower in group R than in group C (14.3 *vs.* 51.4%, *p* < 0.01); and the incidence of nausea, chest congestion, facial flushing, and hypertension were significantly lower in group R than in group C (all *p* < 0.01). Furthermore, the patients’ comfort scores were significantly higher in group R than in group C (8.0 ± 1.8 *vs.* 3.6 ± 2.1, *p* < 0.01).

**Conclusion:**

Our results demonstrate that an intravenous low-dose remifentanil infusion can effectively prevent carboprost-related adverse reactions during cesarean delivery under combined spinal and epidural anesthesia.

**Clinical Trial Registration:**

We pre-registered this study at http://www.chictr.org.cn/showproj.aspx?proj=27707 (ChiCTR1800016292).

## Introduction

Postpartum hemorrhage accounts for almost 20% of maternal mortality cases worldwide (Ducloy-Bouthors et al., 2014; Lockhart, 2015; Henriquez et al., 2018). An increase in the incidence of postpartum hemorrhage (Van Stralen et al., 2016) has been attributed to an increased incidence of uterine atony (Bateman et al., 2010). In the early 1980s, the use of analogs of prostaglandin F2 alpha such as carboprost (Hemabate®) first emerged to treat postpartum hemorrhage, with proven efficacy (Hayashi and Castillo, 1981; Buttino and Garite, 1986), especially in cases of severe postpartum hemorrhage (Bai et al., 2014; Bateman et al., 2014; Butwick et al., 2015). However, prostaglandin F2 alpha analogs may induce a series of adverse reactions, including nausea, vomiting, headaches, diarrhea, facial flushing, and high blood pressure (Buttino and Garite, 1986; Bai et al., 2014). Although these adverse events are generally mild and nonfatal (Buttino and Garite, 1986), they increase the discomfort of parturient patients and adds an unpleasant experience to the patient undergoing cesarean deliveries under spinal-epidural anesthesia. While apart from symptomatic treatment, there are no standard treatments for carboprost side effects.

Studies have shown that remifentanil can be safely used in parturient patients and neonates (Kan et al., 1998; Lee et al., 2017). In our practice, we observed that patients were almost spared all the adverse reactions induced by carboprost when given remifentanil as a form of rescue analgesia during cesarean sections under intraspinal anesthesia. Moreover, the administration of remifentanil reduced the need for additional drugs (which may be harmful to the baby during breastfeeding) that are normally used to treat the adverse reactions induced by carboprost. Combined spinal and epidural anesthesia is the preferred anesthetic technique for cesarean deliveries (Cheesman et al., 2014; Ahiskalioglu et al., 2017; Klimek et al., 2018). Thus, we designed this prospective, randomized controlled trial to explore the effects of an intravenous infusion of low-dose remifentanil on the adverse reactions induced by carboprost during cesarean delivery under combined spinal and epidural anesthesia. Among all adverse reactions induced by carboprost, a reduced incidence of vomiting was set as the primary outcome for this study.

## Materials and Methods

### Study Design

The Ethical Committee of Jiaxing University Affiliated Women and Children Hospital approved the study protocol in May 2018 (TG2018-3), and we obtained signed informed consent in accordance with the Declaration of Helsinki from all participants the day before surgery. For this prospective randomized controlled study, patients who presented for elective cesarean section under combined spinal and epidural anesthesia from May 2018 to November 2018 at the Jiaxing University Affiliated Women and Children Hospital were enrolled.

### Patients

For the analyses, we included patients with American Society of Anesthesiologists (ASA) physical status II or III, aged from 19 to 40 years, with a gestational age ≥37 weeks, who had also received carboprost tromethamine during the cesarean operation. We excluded patients presenting contraindications for combined spinal and epidural anesthesia, and those with anesthesia puncture failure or anesthesia spread level lower than T6. We also excluded patients with severe asthma, a history of allergy to carboprost, opioids, or bupivacaine, or those with significant obstetric morbidity.

### Randomization

An employee who was not involved in the study performed the randomization. Non-transparent, sealed envelopes were prepared and a slip of paper with a computer-generated description of whether the patient should receive the injection of carboprost combined with remifentanil (group R) or carboprost combined with saline (group C) was placed within each envelope. Once the parturient patient needed treatment with carboprost, based on the obstetric evaluation, a nurse who was not involved in the study, opened an envelope and prepared the appropriate procedures accordingly. The patient was blinded to the procedures.

### Standard Protocol

The parturient patients fasted for 8 h and were prohibited from drinking 2 h before the procedure. We established a peripheral intravenous access and administered 8 ml/kg Ringer’s lactate solution before the induction of anesthesia. We performed standard ASA monitoring, including mean arterial pressure (MAP), heart rate (HR), SpO_2_, and respiration rate (RR) measurements, and provided the parturient patients with oxygen through masks (5 L/min) after they were taken to the operating room.

The anesthesiologist administered combined spinal epidurals at the L3/4 interspace using a midline approach, with patients in the right lateral decubitus position. Epidural and spinal punctures were confirmed by a loss of resistance and after obtaining a free flow of cerebrospinal fluid, respectively. After injecting 1.5–2.5 ml of 0.5% hyperbaric bupivacaine (0.75% bupivacaine in 10% glucose) into the subarachnoid space over 10 s, the anesthesiologist inserted an epidural catheter 3 cm cephalad into the epidural space. After these procedures, we promptly placed parturient patients in a supine position with a right pelvic wedge to facilitate left uterine displacement. We initiated the surgery when the anesthesia spread level had reached T6 or above, and administered 0.5% ropivacaine through the epidural catheter for rescue analgesia (repeating the administration if the block level was lower than T6).

After delivery, we administered 20 units of oxytocin by myometrial injection. Subsequently, we started a continuous intravenous infusion of 20 units of oxytocin diluted in 1,000 ml of Ringer’s solution at a rate of about 250 ml/h. The obstetrician evaluated the uterine contractions at that time. A push bolus of 250 µg carboprost (as tromethamine, Hemabate®Pharmacia & Upjohn, Kalamazoo, MI, USA) was administered *via* myometrial injection to patients when needed. Remifentanil (Remifentanil hydrochloride, Rui Jie, Yichang Renfu Pharmaceutical Co., Ltd, China) was administered *via* intravenous target-controlled infusion (1.5 ng/ml target effect-site concentration using the Minto model) once carboprost was administered to the patients in group R. The patients in group C, received remifentanil-equivalent volumes of normal saline instead, with a similar carboprost injection. The same dose of carboprost was repeated if necessary. We continued the remifentanil or saline infusions until completion of the skin sutures. After the procedure, we transported parturient patients to the post-anesthesia care unit (PACU) for at least 30 min.

The same surgical team and the same anesthesiologist performed all procedures. The same anesthesia nurse recorded the characteristics and evaluation indicators of all parturient patients and assessed their comfort score during both surgery and their stay in the PACU using a numerical rating scale (NRS). The anesthesia nurse trained parturient patients the detailed rules regarding the application of the NRS score before entering the operating room. The NRS scores ranged from 0, being very uncomfortable, to 10, being very comfortable. If the visual analog scale (VAS) pain scores were ≥4 points after fetal delivery, we administered a single intravenous dose of 5 µg sufentanil. If vomiting occurred, we administered 5 mg of tropisetron intravenously. We defined hypotension as systolic pressure values <90 mmHg or a decline in systolic pressure >30% and treated it with intravenous ephedrine (5 mg) or neosynephrine (100 µg) as needed. We defined bradycardia as HR values <55 beats/min and treated this with intravenous atropine (0.5 mg). We treated hypoxemias with an SpO_2_ <95%, by stopping the administration of any opiates. We used warming systems for shivering parturient patients.

### Data Collection

We recorded the maximal VAS score and minimum SpO_2_during surgery. We recorded and considered the incidence of carboprost-related adverse reactions, such as vomiting, nausea, chest congestion, facial flushing, hypertension, tachycardia, cough, and shivering during surgery and PACU stay for the final analysis. We recorded the RR, HR, and MAP before anesthesia (T0), 1 min before the carboprost injection (T1), 1 min after the carboprost injection (T2), 5 min after the carboprost injection (T3), 15 min after the carboprost injection (T4), 30 min after the carboprost injection (T5), and 1 h after the carboprost injection (T6). We also recorded the demographic variables of all parturient patients, including the ASA status, age, height, weight, and pregnancy information.

### Statistical Analysis

We established a reduced incidence of vomiting as the primary outcome for this study. In a preliminary trial with 22 patients in each group, the incidence of vomiting was 18.2 and 54.5% in groups R and C, respectively. Based on these preliminary results, we calculated the need to include 32 parturient patients in each group for a 90% power, to detect a 60% reduction in the incidence of vomiting at the 5% significance level (two-tailed). Considering the possibility of dropouts, we aimed to recruit 35 parturient patients in each group.

We presented the data as means ± SDs, medians (quartile), or numbers (%) as appropriate. We used the IBM SPSS Statistics for Windows 22.0 software (IBM Corp, Armonk, NY, USA) to analyze the data, using the independent *t-*test, rank sum test, chi-squared test, chi-squared test with continuity correction, and the repeated measured analysis of variance, as appropriate. We considered *p* values <0.05 as statistically significant.

## Results

**Figure 1:**
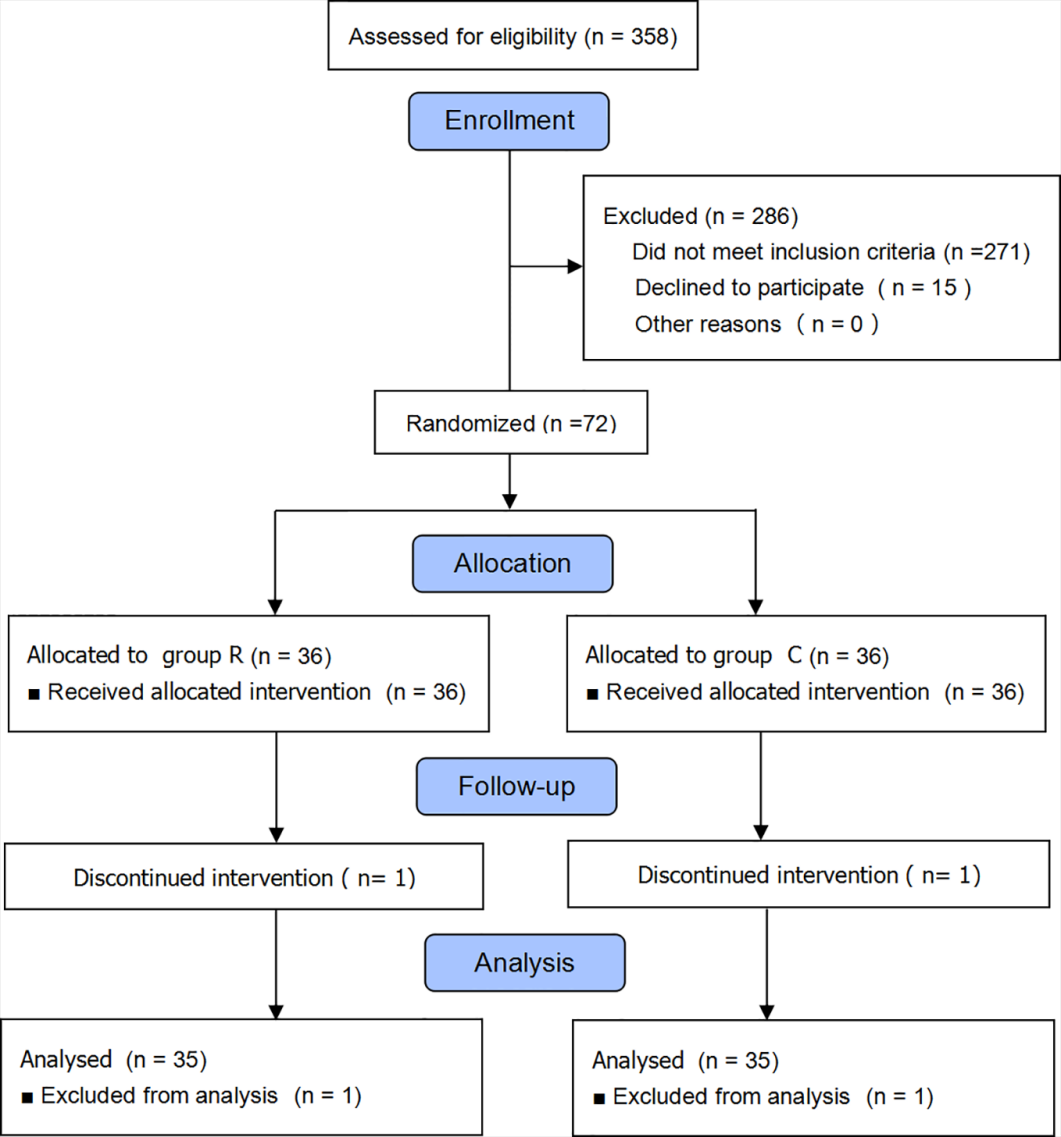
Consolidated Standards of Reporting Trials (CONSORT) flow diagram.

We initially enrolled 358 parturient patients and eventually included 72, but excluded two from the data analysis because they declined to participate (**Figure 1**). We found no differences between the two groups in terms of maternal and newborn baseline characteristics (all *p* > 0.05); see **Table 1**.

**Table 1:** Maternal and newborn characteristics.

	Group C (n = 35)	Group R (n = 35)	*P* value
ASA status (II/III), n	29/6	30/5	0.912^*^
Age, years	29.8 ± 4.6	29.0 ± 4.8	0.418**^#^**
Height, cm	160.7 ± 4.2	160.4 ± 4.9	0.834**^#^**
Weight, kg	76.1 ± 9.5	72.2 ± 10.1	0.097**^#^**
Singleton/twin pregnancy, n	32/3	31/4	0.909**^&^**
Gestational age, weeks	37.9 ± 1.3	38.1 ± 1.2	0.865**^#^**
Neonatal weight, kg	3.9 ± 0.8	3.9 ± 0.9	0.874**^#^**

Data are expressed as mean ± standard deviation, unless otherwise indicated; ASA, American Society of Anesthesiologists; ^*^p was tested with the chi-squared test; **^#^**p was tested with the independent t-test; **^&^**p was tested with the chi-squared test with continuity correction.

All patients received a single injection of 250 µg of carboprost. We observed eight types of adverse reactions. The incidence of vomiting (the primary outcome) was significantly lower in group R than in group C (14.3 *vs.* 51.4%, *p* < 0.01); and the incidence of nausea, chest rigidity, facial flushing, and hypertension were also lower in group R (all *p* < 0.01). The rates of tachycardia, cough, and shivering were similar between the two groups (all *p* > 0.05); see **Table 2**.

**Table 2:** Incidence of carboprost-related adverse reactions.

	Group C (n = 35)	Group R (n = 35)	*P* value
Vomiting, n (%)	18 (51.4%)	5 (14.3%)	<0.01^*^
Nausea, n (%)	29 (82.9%)	11 (31.4%)	<0.01^*^
Chest rigidity, n (%)	28 (80.0%)	5 (14.3%)	<0.01^*^
Flushed face, n (%)	13 (37.1%)	1 (2.9%)	<0.01^*^
Hypertension, n (%)	11 (31.4%)	0 (0.0%)	<0.01^*^
Tachycardia, n (%)	14 (40.0%)	6 (17.1%)	0.074^*^
Cough, n (%)	3 (8.6%)	3 (8.6%)	1.000**^&^**
Shivering, n (%)	4 (11.4%)	1 (2.9%)	0.180**^&^**

Data are presented as number (percentage); ^*^p was tested with the chi-squared test; **^&^**p was tested with the chi-squared test with continuity correction.

**Figure 2:**
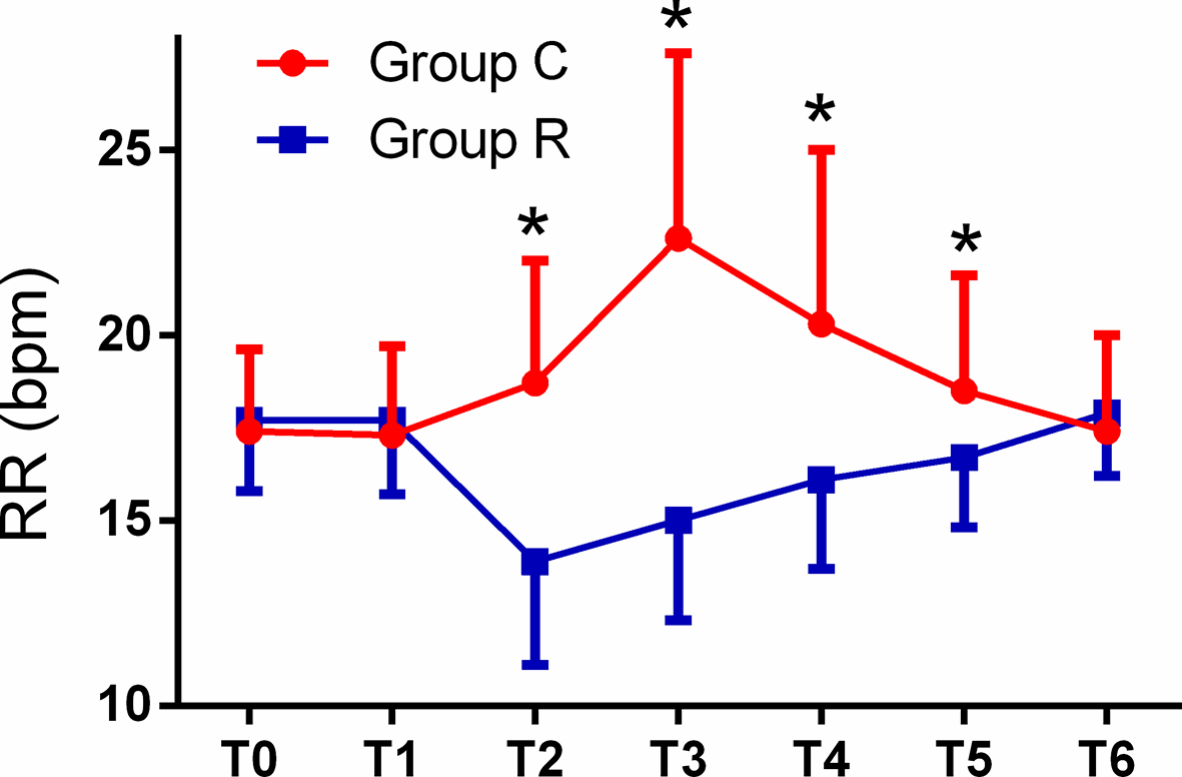
Comparison of respiratory rates (RRs) from T0 to T6 between the two groups. The RRs in the remifentanil group (group R) were significantly lower at T2, T3, T4, and T5, as compared with corresponding values in the control (group C) (all *p < 0.01).

The patient comfort scores, which were measured using the NRS were significantly better in group R than in group C (8.0 ± 1.8 *vs.* 3.6 ± 2.1, *p* < 0.01), and the frequency of tropisetron use and maximal VAS scores were significantly lower in group R than in group C (both *p* < 0.01). Although the mean minimum RR was significantly lower in group R (*p* < 0.01), we found no significant differences in the mean minimum SpO_2_ between the groups (both *p* > 0.05). The mean RRs were significantly lower at T2, T3, T4, and T5 in group R than in group C (all *p* < 0.01). The mean HRs were significantly higher at T3, T4, and T5 in group C than in group R (*p* < 0.01, or *p* < 0.05), while the mean HRs in both groups at all time points were within normal levels. The mean MAP was significantly higher at T3 in group C in group R (*p* < 0.01); see **Table 3** and **Figures 2** **–** **4**.

**Table 3:** Maternal and newborn surgical details.

	Group C (n = 35)	Group R (n = 35)	*P* value
Duration of surgery, min	55.3 ± 17.3	52.1 ± 12.9	0.251**^#^**
Blood loss during surgery, ml	300 [300,400]	350 [300,400]	0.439**^$^**
Rescue analgesia with ropivacaine, n (%)	11 (31.4%)	9 (25.7%)	0.655^*^
Remifentanil dosage, μg	0	212.4 ± 94.2	<0.01**^#^**
Rescue analgesia with sufentanil, n (%)	8 (22.9%)	0	<0.01**^&^**
Use of tropisetron, n (%)	19 (54.3%)	5 (14.3%)	<0.01^*^
Maximal VAS score	0 [0, 3]	0 [0, 0]	<0.01**^$^**
Minimum RR, bpm	17.5 ± 3.8	13.0 ± 2.6	<0.01**^#^**
Minimum SpO_2_, (%)	96.9 ± 1.6	97.0 ± 2.1	0.800**^#^**
Patients’ comfort score with NRS measurement	3.6 ± 2.1	8.0 ± 1.8	<0.01**^#^**

Data are presented as mean ± standard deviation, median (quartile), or number (percentage). VAS, visual analog scale; RR, respiratory rate; SpO_2_, peripheral oxygen saturation; NRS, numerical rating scale; ^*^p was tested with the chi-squared test; **^#^**p was tested with the independent t-test; **^&^**p was tested with the chi-squared test with continuity correction; **^$^**p was tested with the rank sum test.

**Figure 3:**
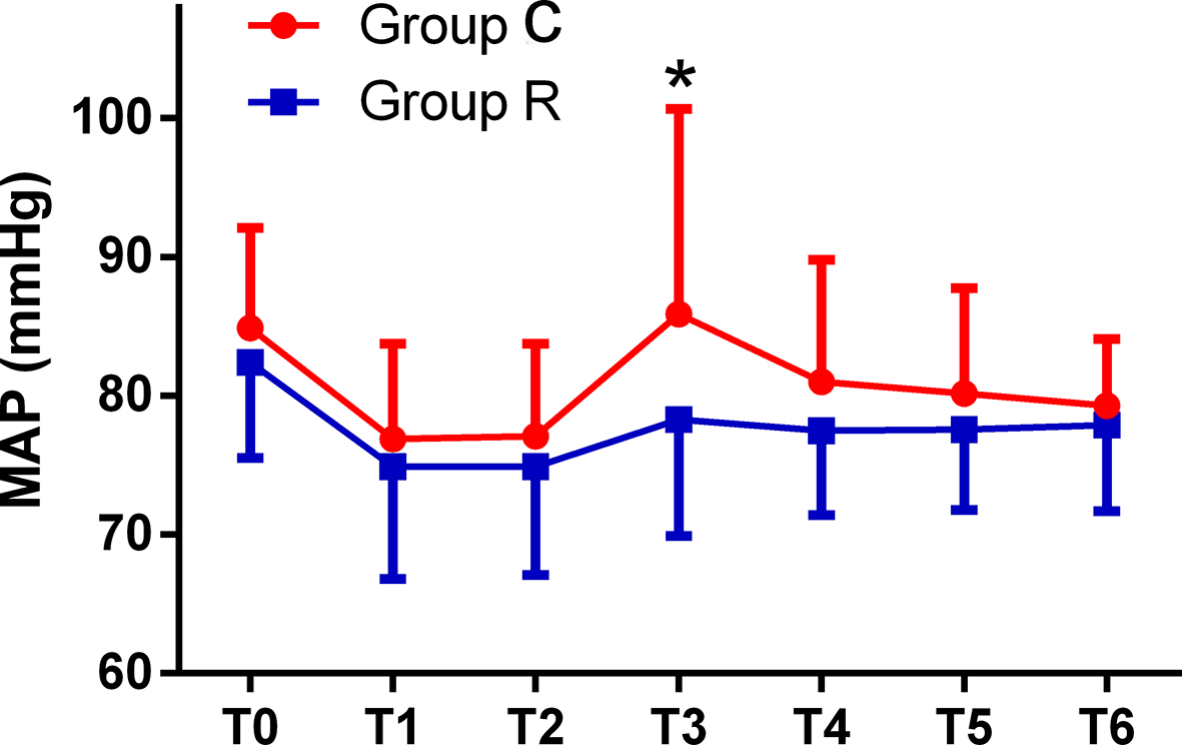
Comparison of mean arterial pressures (MAPs) from T0 to T6 between the two groups. The MAPs were significantly higher at T3 in the control (group C) than at T3 in the remifentanil group (group R) (*p < 0.01).

**Figure 4:**
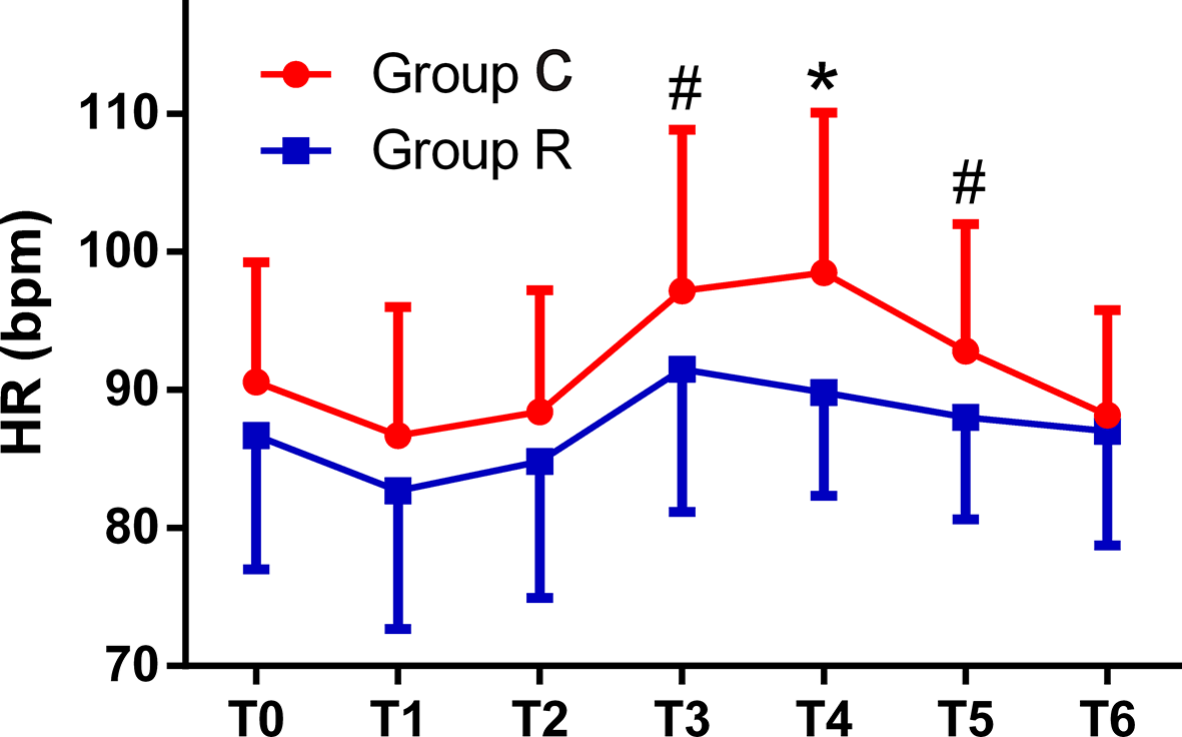
Comparison of heart rates (HRs) from T0 to T6 between the two groups. The HRs were significantly higher at T3, T4, and T5 in the control (group C) as compared with corresponding values in the remifentanil group (group R) (*p < 0.01 or **^#^***p* < 0.05).

## Discussion

These results demonstrate that intravenous infusions of low-dose remifentanil can effectively alleviate adverse reactions induced by myometrial injection of carboprost during cesarean delivery under combined spinal and epidural anesthesia. Maternal comfort was significantly improved without any obvious complications.

Carboprost, as a second-line drug, has been considered for both prophylaxis and the treatment of postpartum hemorrhage (Heesen et al., 2019). It is the synthetic 15-methyl analog of prostaglandin F_2α_ that induces smooth muscle contraction and causes prostaglandin-like complications, including vomiting, nausea, chest congestion, diarrhea, headaches, hypertension, and bronchial asthma (Bai et al., 2014). In the present study, parturient patients administered remifentanil, experienced a lower incidence of vomiting after carboprost administration than those not given remifentanil. We also observed reductions in the incidence of most of the other carboprost-induced adverse reactions, including nausea, chest rigidity, facial flushing, and hypertension.

In the current study, the low incidence of nausea, vomiting, and chest congestion in patients administered remifentanil was due to its inhibitory effects on carboprost-induced smooth muscle contraction (Holzer, 2009) as well as its sedation effects (Kisilewicz et al., 2017). In the clinic, we observed that the remifentanil’s antiemetic benefit was better than usual antiemetics given at the time of carboprost because the rapid onset of remifentanil and latency effect of usual antiemetics. At the same time, routine prophylactic administration of antiemetics during cesarean section under spinal anesthesia is not indicated, and the use of these drugs should be in accordance with assumed safety for mother and baby (Balki and Carvalho, 2005). Inhibition of the sympathetic nervous system by remifentanil may reduce the incidence of flushing, hypertension, and tachycardia in parturient patients treated with carboprost. Studies have shown that remifentanil may increase the incidence of postoperative shivering (Nakasuji et al., 2010; Hoshijima et al., 2016). However, in the present study, we observed no differences in the incidence of shivering between groups. This might have been due to the low remifentanil doses administered.

In the present study, we administered remifentanil to parturient patients after delivery of the fetus and the administration of carboprost. The pain and discomfort caused by cesarean sections is mainly due to uterine traction and peritoneal sutures after delivery of the fetus. In parturient patients treated with remifentanil, the need for rescue analgesia (with sufentanil) and the maximal VAS scores were both reduced. The sedation and analgesic effects of remifentanil (Kisilewicz et al., 2017) and the reduced incidence of carboprost side effects contributed to improved comfort and satisfaction of the mothers in group R compared with those in group C. Furthermore, the mean minimum RRs and the mean minimum SpO_2_ were both within normal ranges. Studies have shown that remifentanil can be safely used during labor analgesia, with few side effects on the fetus (Ohashi et al., 2016; Wilson et al., 2018). Moreover, the need for additional drugs to treat carboprost-induced adverse reactions (which may be harmful to the baby during breastfeeding) was reduced. Thus, remifentanil administration can maximize maternal comfort without increasing side effects in the newborns.

In the present study, differences in hemodynamic and respiratory-related parameters were statistically significant between the two groups at some time points, but almost all parameters were within normal ranges at all time points. Remifentanil is a potent opioid analgesic with an ultra-short-acting pharmacokinetic profile (Komatsu et al., 2007). The quick onset and offset of its effects make the use of remifentanil convenient, even within the context of different surgical stimuli, and permits rapid recovery after withdrawal (Komatsu et al., 2007).

Carboprost consistently reaches peak plasma concentration 0.5 h after intramuscular injection at a dose of 250 µg. In the current study, when considering the RR, HR, and MAP, prostaglandin-like side effects mainly occurred within 30 min after the administration of carboprost and gradually disappeared within 1 h. Thus, the RR, HR, and MAP returned to baseline 1 h after carboprost administration, and no significant differences in these parameters were observed between the two groups. It is worth mentioning that the respiratory depression caused by remifentanil was not observed in this study. Furthermore, the SpO_2_ showed no significant differences between the two groups at any time point.

We are aware of some limitations in the present study. Another study has shown that remifentanil may decrease contractions of the myometrium in pregnant rats (Kayacan et al., 2007). In the present study, although we found no differences in blood loss during cesarean sections between groups, we cannot rule out the effects of remifentanil on uterine smooth muscle contractions, which could partially offset the therapeutic effects of carboprost. Whether the dosage of carboprost should be increased or not, when combined with remifentanil, needs further study. In addition, Liu et al. (2015) reported that dexmedetomidine can also inhibit the adverse reactions of carboprost during cesarean sections, and suggested that other studies should determine whether remifentanil or dexmedetomidine is superior for such purposes. In addition, ours was a relatively small study population; thus, some complications caused by remifentanil may not have been observed. Furthermore, we used only a target effect-site remifentanil concentration of 1.5 ng/ml. Whether this is the most suitable dose or not is unknown.

In conclusion, our results demonstrate that an intravenous target-controlled infusion of 1.5 ng/ml of target effect-site remifentanil concentration can effectively prevent the adverse reactions induced by carboprost during cesarean delivery under combined spinal and epidural anesthesia.

## Data Availability Statement

The datasets analyzed in this article are not publicly available. Requests to access the datasets should be directed to jxxmxy@163.com.

## Ethics Statement

The studies involving human participants were reviewed and approved by The Ethical Committee of Jiaxing University affiliated Women and Children Hospital. The patients/participants provided their written informed consent to participate in this study.

## Author Contributions

C-NW and Q-HZ conceived the idea and conducted the experiments. Q-HZ wrote the manuscript with support from C-NW and J-HD. X-YC helped in analyzing the data. All authors contributed to the article and approved the submitted version.

## Funding

The Technology Bureau of Jiaxing, Zhejiang province (2015C23023), and the Health Development Planning Commission of the Zhejiang province (2015KYB389) provided funding for this work.

## Conflict of Interest

The authors declare that the research was conducted in the absence of any commercial or financial relationships that could be construed as a potential conflict of interest.
